# Development and external validation of a model for post-endoscopic retrograde cholangiopancreatography pancreatitis

**DOI:** 10.1016/j.isci.2025.112570

**Published:** 2025-05-02

**Authors:** Gang Wang, Qikai Sun, Hai Zhu, Song Qiao, Peng Xu, Xiangyu He, Xiangkun He, Xiaosi Hu, Mingming Song, Qiuyan Zhang, Zhenyu Feng, Yue Chen, Yue Gao, Zhiyuan Jin, Wen Li, Haizheng Tang, Chaoqun Yan, Yajun Wei, Shibo Xu, Gang Hu, Xiuhua Zhang, Jinxin Zheng, Cheng Wang

**Affiliations:** 1 Department of General Surgery, The First Affiliated Hospital of USTC, Division of Life Sciences and Medicine, University of Science and Technology of China, Hefei, Anhui 230031, China; 2 Department of General Surgery, The First Affiliated Hospital of Anhui Medical University, Hefei, Anhui 230001, China; 3 Department of Outpatient, The First People’s Hospital of Hefei, Hefei, Anhui 230031, China; 4 Department of Hepatobiliary and Pancreatic Surgery, Northern Jiangsu People’s Hospital, Clinical Medical College of Yangzhou University, Yangzhou, Jiangsu 225001, China; 5 Department of Hepatobiliary Surgery, Hunan Provincial People’s Hospital, the First Affiliated Hospital of Hunan Normal University, Changsha, Hunan 410000, China; 6 Department of General Surgery, No. 904 Hospital of the PLA Joint Logistics Support Force, Wuxi, Jiangsu 214044, China; 7 Department of General Surgery, Anhui No.2 Provincial People’s Hospital, Hefei, Anhui 230041, China; 8 Department of General Surgery, The First Affiliated Hospital of Bengbu Medical College, Bengbu, Anhui 233000, China; 9 Department of General Surgery, The Second People’s Hospital of Hefei, Hefei, Anhui 230011, China; 10 Department of Gastroenterology, The Second Affiliated Hospital of Nanjing Medical University, Nanjing, Jiangsu 210011, China; 11 Department of Hepatobiliary Surgery, The Second Affiliated Hospital of Soochow University, Suzhou, Jiangsu 215006, China; 12 Department of Gastroenterology, Xuzhou Central Hospital, Xuzhou, Jiangsu 221009, China; 13 Department of Pediatric Surgery, The First Affiliated Hospital of Wannan Medical College, Wuhu, Anhui 246400, China; 14 Endoscopy Center Department, The First Affiliated Hospital of USTC, Division of Life Sciences and Medicine, University of Science and Technology of China, Hefei, Anhui 230031, China; 15 School of Global Health, Chinese Center for Tropical Diseases Research-Shanghai Jiao Tong University School of Medicine, Shanghai 200025, China

**Keywords:** Public health, Bioinformatics, Machine learning

## Abstract

Post-endoscopic retrograde cholangiopancreatography (ERCP) pancreatitis (PEP) is a common complication in patients undergoing ERCP for choledocholithiasis, yet effective predictive models are lacking. This study included 2,247 patients who underwent ERCP for complete stone removal at the First Affiliated Hospital of USTC from January 2015 to January 2023. Six machine learning algorithms were utilized, incorporating 25 clinical parameters, to develop a predictive model for PEP risk. The random forest (RF) algorithm achieved the highest accuracy, with an area under the receiver operating characteristic curve (AUC) of 0.947 in the internal dataset. Key risk factors for PEP identified include difficult cannulation, a history of pancreatitis, smaller common bile duct diameter, and female gender. Validation with datasets from 12 external centers showed AUC values ranging from 0.576 to 0.913, with an average of 0.768. An interactive R Shiny web application was also developed, offering a user-friendly tool for predicting PEP risk and enabling individualized management.

## Highlights

• First externally validated machine learning model for predicting ERCP pancreatitis risk
• Model shows superior accuracy in identifying high-risk patients
• Tool helps clinicians evaluate pancreatitis risk after ERCP for choledocholithiasis

## Graphical abstract

**Figure:**
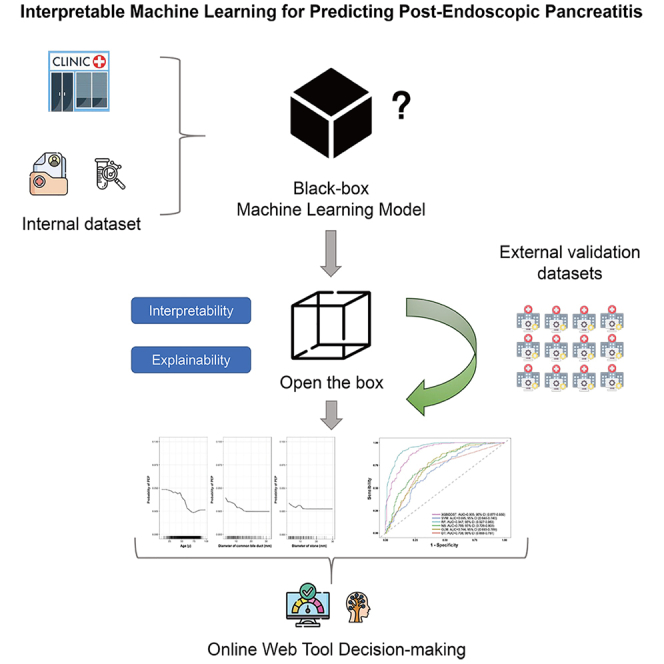

## Introduction

Choledocholithiasis, commonly referred to as common bile duct stones (CBDS), represents a globally prevalent chronic and recurrent gastrointestinal disorder. Endoscopic retrograde cholangiopancreatography (ERCP) remains the cornerstone in the management of choledocholithiasis, lauded for its safety, efficacy, minimal invasiveness, expedited patient recovery, and reduced complication rates.^1^ However, post-ERCP pancreatitis (PEP) emerges as the most common and grave adverse event, manifesting in approximately 2–9% of cases, a proportion that can escalate beyond 25% in specific high-risk cohorts.^2^^,^^3^^,^^4^ The etiological landscape of PEP is multifactorial, broadly categorized into patient-specific and procedural risk elements. Patient-related factors encompass demographic and clinical characteristics, such as female gender, younger age, suspected sphincter of Oddi dysfunction (SOD), and a history of pancreatitis. Concurrently, procedural determinants include challenges in cannulation, inadvertent guidewire insertion into the pancreatic duct, among others.^5^

Currently, the prediction of PEP risk following complete stone clearance in choledocholithiasis patients remains challenging due to limited data on complete stone extraction via ERCP, the relatively low incidence of PEP, and the presence of numerous confounding factors.^6^^,^^7^ This study employed six distinct machine learning algorithms to identify the most effective predictive model, rigorously validated through both internal and external assessments. It specifically investigates the intricate correlations between the clinical characteristics of patients with CBDS and the onset of PEP. The resultant model, demonstrating robust predictive capability, has been integrated into a user-friendly online platform. This innovation empowers clinicians to devise more targeted and effective management strategies for patients identified as having an elevated risk of developing PEP.

## Results

### Demographic and clinical profiles of patients post-complete ERCP clearance for choledocholithiasis

In this analysis of 2,247 patients with CBDSs, demographic and clinical characteristics were systematically compiled and are detailed in Table 1. The occurrence of PEP in this cohort was 3.38% (76 out of 2,247 patients), with a female-to-male ratio of 2.30:1 (53 females vs. 23 males) within the PEP subgroup. Notably, patients who developed PEP were significantly younger on average than those who did not (mean age 54.47 ± 13.26 years vs. 60.07 ± 16.01 years, *p* = 0.003). Additionally, the incidence of PEP was markedly higher in patients with a history of pancreatitis (18.42% vs. 4.97%, *p* = 0.001), those with a smaller diameter of the common bile duct (mean diameter 9.21 ± 2.72 mm vs. 10.92 ± 4.44 mm, *p* = 0.001), those with smaller bile duct stones (mean diameter 6.28 ± 2.64 mm vs. 7.22 ± 3.61 mm, *p* = 0.021), those experiencing difficult cannulation (25.00% vs. 6.86%, *p* < 0.001), and those who underwent EST (55.26% vs. 42.93%, *p* = 0.044).

**Table 1:** Clinical demographics and characteristics of cohort in PEP patients with CBDSs

Variables		All patients (2247)	Non-PEP (2171)	PEP (76)	P
Gender	Male	1054 (46.91)	1031 (47.49%)	23 (30.26%)	0.004
	Female	1193 (53.09)	1140 (52.51%)	53 (69.74%)	
Age (year)		59.88 (15.96)	60.07 (16.01)	54.47 (13.26)	0.003
Height (cm)		163.04 (8.46)	163.07 (8.49)	162.07 (7.44)	0.309
Weight (kg)		62.02 (11.19)	62.01 (11.22)	62.18 (10.52)	0.896
HBP	NO	1560 (69.43)	1509 (69.51%)	51 (67.11%)	0.749
	YES	687 (30.57)	662 (30.49%)	25 (32.89%)	
DM	NO	1961 (87.27)	1890 (87.06%)	71 (93.42%)	0.144
	YES	286 (12.73)	281 (12.94%)	5 (6.58%)	
History of pancreatitis	NO	2125 (94.57)	2063 (95.03%)	62 (81.58%)	<0.001
	YES	122 (5.43)	108 (4.97%)	14 (18.42%)	
History of hepatitis	NO	2198 (97.82)	2123 (97.79%)	75 (98.68%)	0.900
	YES	49 (2.18)	48 (2.21%)	1 (1.32%)	
WBC (10^9^/L)		6.04 (2.72)	6.06 (2.73)	5.52 (2.57)	0.094
N (10^9^/L)		3.87 (2.65)	3.89 (2.66)	3.36 (2.52)	0.090
HB (g/L)		127.98 (16.62)	127.92 (16.69)	129.49 (14.52)	0.420
PLT (10^9^/L)		203.92 (75.56)	204.25 (75.86)	194.47 (66.14)	0.267
TB (umol/L)		35.33 (49.65)	35.32 (48.65)	35.57 (73.06)	0.966
ALT (U/L)		121.31 (169.47)	122.31 (170.35)	92.86 (139.85)	0.136
AST (U/L)		85.00 (129.48)	85.88 (130.69)	59.93 (85.19)	0.086
Diameter of common bile duct (mm)		10.86 (4.40)	10.92 (4.44)	9.21 (2.72)	0.001
Diameter of the largest stone (mm)		7.18 (3.59)	7.22 (3.61)	6.28 (2.64)	0.021
Presence of the gallbladder	NO	1370 (60.93)	1315 (60.57%)	54 (71.05%)	0.085
	YES	877 (39.07)	856 (39.43%)	22 (28.95%)	
With gallstone	NO	1631 (72.54)	1568 (72.22%)	62 (81.58%)	0.096
	YES	616 (27.46)	603 (27.78%)	14 (18.42%)	
With intrahepatic bile duct stones	NO	2061 (91.72)	1990 (91.66%)	71 (93.42%)	0.738
	YES	186 (8.28)	181 (8.34%)	5 (6.58%)	
Number of Stones	1	956 (42.55)	919 (42.33%)	37 (48.68%)	0.531
	2	148 (6.59)	144 (6.63%)	4 (5.26%)	
	multiple	1143 (50.87)	1108 (51.04%)	35 (46.05%)	
Position relationship between papilla and diverticula	In	103 (4.58)	102 (4.70%)	1 (1.32%)	0.351
	Next	675 (30.04)	653 (30.08%)	22 (28.95%)	
	Non-diverticula	1469 (65.38)	1416 (65.22%)	53 (69.74%)	
Difficult cannulation	NO	2079 (92.52)	2022 (93.14%)	57 (75.00%)	<0.001
	YES	168 (7.48)	149 (6.86%)	19 (25.00%)	
EST	NO	1273 (56.65)	1239 (57.07%)	34 (44.74%)	0.044
	YES	974 (43.35)	932 (42.93%)	42 (55.26%)	
EPBD	NO	555 (24.70)	530 (24.41%)	25 (32.89%)	0.121
	YES	1692 (75.30)	1641 (75.59%)	51 (67.11%)	

HBP, Hypertension; DM, Diabetes Mellitus; WBC, White blood cell; N, Neutrophil; HB, Hemoglobin; PLT, Platelet; TB, Total bilirubin; ALT, Alanine transaminase; AST, Aspartate transaminase; EST, Endoscopic sphincterotomy; EPBD, Endoscopic papillary balloon dilation.

### Correlations between categorical and continuous variables in relation to PEP incidence

**Figure 1:**
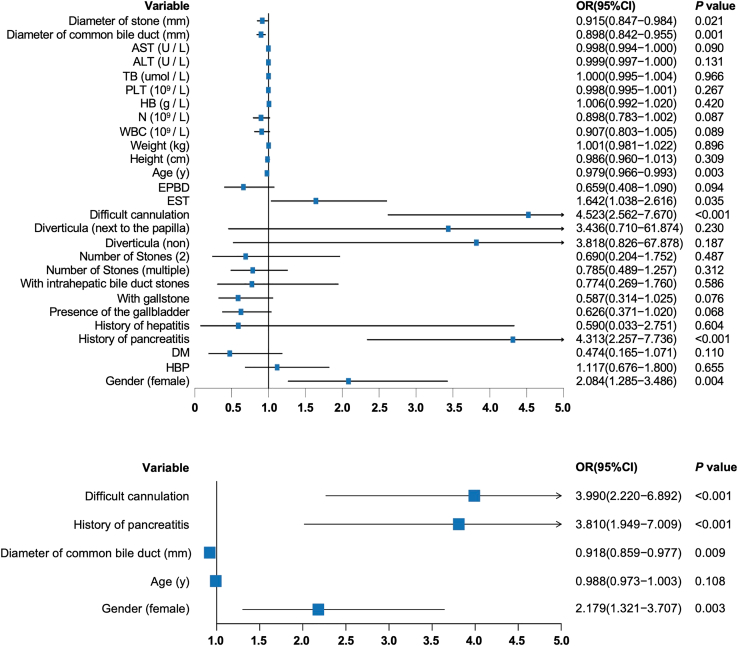
The forest plot of univariate and multivariate logistic regression analyses

**Figure 2:**
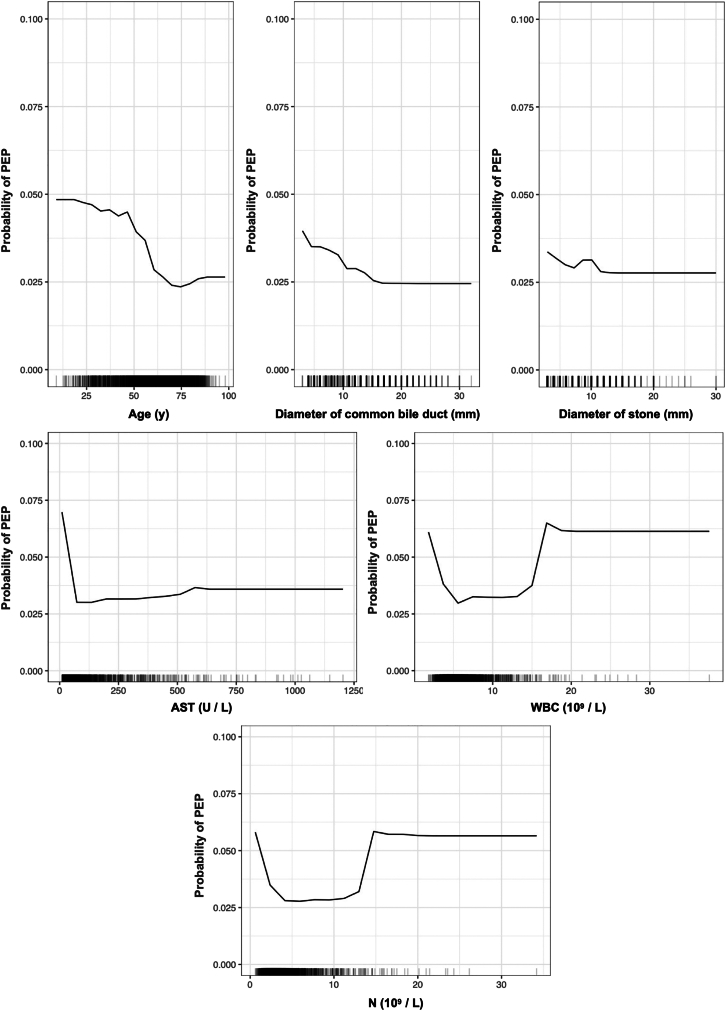
The partial dependence plots for the top six variables in the predictive model

Figure 1 delineates several independent risk factors for the development of PEP following CBD stone clearance. Notably, difficult cannulation, a history of pancreatitis, a smaller diameter of the common bile duct, and female gender are identified as independent predictors. Among these, difficult cannulation presents the highest odds ratio (OR) of 3.990 (95% confidence interval [CI] = 2.220–6.892). PDPs were utilized to examine the impact of variables, such as age, common bile duct diameter, and stone diameter (selected based on *p* < 0.05) on PEP risk, controlling for marginal effects in the model. As depicted in Figure 2, a notable trend is observed wherein PEP risk decreases with increasing age up to 42 years. A slight uptick in risk is seen between ages 42–46 years, followed by a sharp decline, reaching a minimum at 75 years. In terms of common bile duct diameter, the risk of PEP shows a downward trend between diameters of 3.00 and 16.74 mm, then plateaus. Interestingly, stones measuring 3 mm in the common bile duct present the highest PEP risk. A transient increase in risk is noted for stone diameters between 7.26 and 12.95 mm, beyond which the risk does not significantly change with further increases in stone diameter. When variables with *p* ≤ 0.10 are included in the PDP analysis, additional factors, such as aspartate transaminase (AST), WBC count, and N value are considered. The risk of PEP gradually decreases as AST levels range from low to 72.79 U/L and then generally stabilizes. Moreover, PEP risk is lower when WBC levels are between 5.59 and 14.99 (×10^9^/L) or N levels are between 4.14 and 12.98 (×10^9^/L). However, significant increases in PEP risk are observed when WBC and N values exceed or fall below these specified ranges.

### Development and validation of a machine learning model for PEP

**Figure 3:**
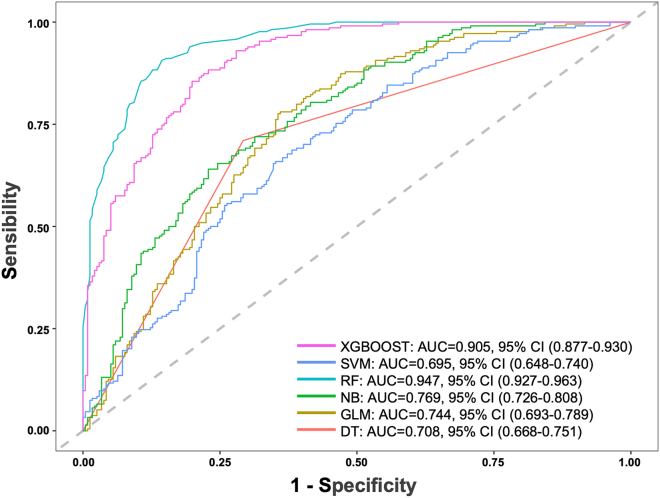
ROC curves for six different machine learning models in the internal dataset

**Figure 4:**
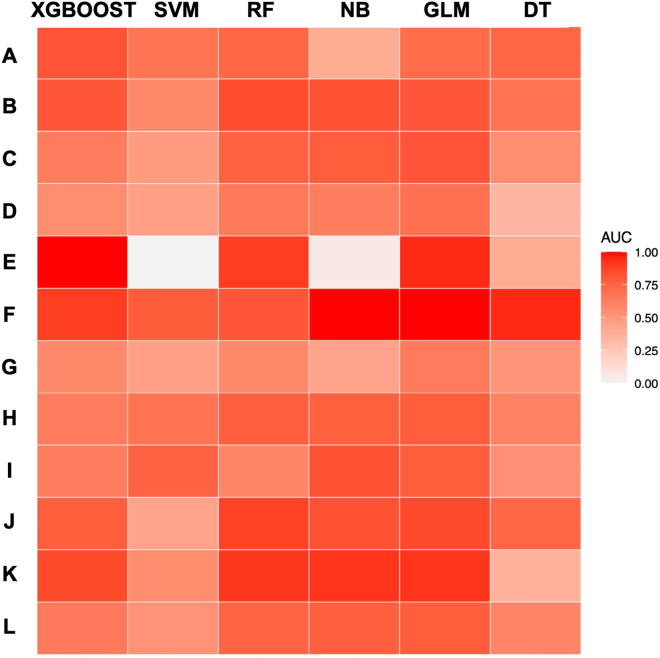
The heatmap of the AUC scores across 12 external center dataset

This study utilized a set of specified variables to assess the accuracy of PEP prediction in patients undergoing ERCP, employing six different machine learning algorithms. The receiver operating characteristic curve (ROC) presented in Figure 3, illustrates the area under the receiver operating characteristic curve (AUC) for the classifiers. The AUC values for the extreme gradient boosting (XGBOOST), support vector machine (SVM), random forest (RF), naive bayes (NB), generalized linear models (GLM), and decision trees (DT) algorithms on the internal test set were 0.905 (95% CI = 0.877–0.930), 0.695 (95% CI = 0.648–0.740), 0.947 (95% CI = 0.927–0.963), 0.769 (95% CI = 0.726–0.808), 0.744 (95% CI = 0.693–0.789), and 0.708 (95% CI = 0.668–0.751) respectively, with the RF algorithm demonstrating the highest prediction performance. When these six models were applied to 12 external datasets without retraining, the heatmap in Figure 4 depicts their predictive performance. The RF algorithm consistently showed robust prediction capabilities across the external institutions, with AUC values ranging from 0.576 to 0.913 and an average of 0.768. This aligns with the internal validation results. Notably, only one institution exhibited an AUC below 0.60, while five institutions achieved an AUC of 0.80 or higher. The comprehensive results of this external validation are detailed in Table S1.

### Development of an online web application for clinical utility

**Figure 5:**
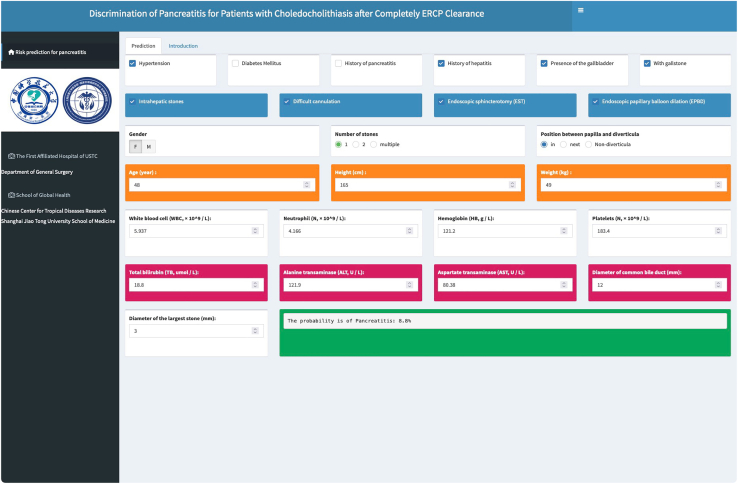
Risk prediction application screen

To enhance the practical application of our predictive model, a web-based risk assessment tool, grounded in the RF model incorporating the aforementioned 25 clinical variables, has been developed. Accessible at https://jamesjin63.shinyapps.io/Shiny_PEP_USTC/, this online calculator provides a user-friendly and freely accessible global platform for real-time assessment of PEP risk. A comprehensive flow chart detailing the operational workflow of this tool is illustrated in Figure 5, elucidating its functionality and ease of use for clinicians worldwide.

## Discussion

Pancreatitis represents the most frequent complication following ERCP, predominantly manifesting in a mild form, though a minority of cases may escalate to severe levels. The exact pathophysiological mechanisms underpinning PEP remain incompletely understood. Potential causes include tissue thermal injury from electrocoagulation during papillotomy, papillary edema due to repeated cannulation, or impaired pancreatic juice drainage from other factors. In our study, the incidence of PEP was 3.38%, aligning with findings from Cotton PB, a notable figure in the field of ERCP.^8^ The demographic profile indicated a higher PEP incidence in younger and female patients. This demographic may exhibit heightened sensitivity to ERCP-related stimuli, corroborating observations noted in both the American Society for Gastrointestinal Endoscopy (ASGE) and European Society of Gastrointestinal Endoscopy (ESGE) guidelines.^5^^,^^9^

In addition to the demographic characteristics identified, our multivariate analysis indicated that difficult cannulation, a history of pancreatitis, and a smaller diameter of the common bile duct are independent risk factors for the development of PEP. These findings are largely in agreement with existing guidelines.^5^^,^^9^ Additionally, PDPs were utilized to analyze the relationship between these identified continuous variables and PEP with greater precision. The PDPs revealed that the relationship between the explanatory variables and PEP is not strictly linear. Notably, the overall risk of PEP decreases with age. However, the decline in risk becomes more pronounced after the age of 46 compared to before 42, reaching its lowest point at 75 years. This trend could be attributed to changes in immune and inflammatory responses in older patients, particularly as immune resistance tends to diminish beyond 75 years. Regarding the diameter of the common bile duct, while there is an overall downward trend in PEP risk, the risk does not further increase beyond a diameter of 16.74 mm. Interestingly, the highest incidence of PEP was observed in cases of choledocholithiasis with stones measuring 3 mm or resembling sediment. This could be due to the propensity of small stones to obstruct the pancreatic duct. There is also a noticeable transient increase in PEP risk for stone diameters ranging from 7.26–12.95 mm, suggesting a multifactorial risk profile. Furthermore, it appears plausible that a heightened immune response from the liver, leukocytes, or neutrophils occurs when patient levels reach an AST level of 72.79 U/L, a WBC count of 5.59–14.99 (×10^9^/L), and neutrophil counts between 4.14 and 12.98 (×10^9^/L).

In a notable international, multicenter, prospective study involving 1,150 patients, the incidence of PEP was documented at 6.1%, with the GB machine learning model yielding a predictive probability of 0.671 for PEP.^10^ Another retrospective study, encompassing 3,362 patients and reporting a PEP incidence of 3.2%, demonstrated an AUC of 0.81 (95% CI: 0.77–0.86) for the optimism-corrected logistic ROC curve.^11^ However, to date, there has been no established predictive model specifically for PEP in patients with complete choledocholithiasis clearance, nor a machine learning model validated across multiple centers. In our study, we addressed this gap by employing 25 clinical parameters to construct six different machine learning models. In the context of this study, which included an internal model comprising 2,247 patients, the RF algorithm demonstrated an impressive predictive accuracy rate of 0.947 for PEP, surpassing other algorithms, such as XGBOOST, SVM, NB, GLM, and DT. Further, when applied across 12 external institutions, the RF algorithm consistently exhibited robust predictive performance, with AUC values ranging from 0.576 to 0.913 and an average of 0.768. These results strongly suggest the reliability and generalizability of the RF model. Given its demonstrated efficacy, we posit that the RF algorithm holds significant promise for predicting PEP in multicenter settings, particularly in patients who have undergone complete stone clearance via ERCP.

Machine learning presents significant advantages over logistic regression for PEP prediction, including its ability to capture complex, non-linear relationships and feature interactions without manual specification, handle high-dimensional data, and provide superior predictive performance.^12^ These models excel in adapting to new information, allowing for continuous improvement and personalization of risk assessments. The RF algorithm stands out in machine learning for its high accuracy, robustness to noise, and versatility in handling both classification and regression tasks across high-dimensional data. It achieves superior predictive performance by aggregating multiple decision trees, reducing the risk of overfitting and ensuring stability in predictions.

Machine learning has revolutionized risk assessment by enabling personalized evaluations based on individual patient characteristics. This approach to decision-making is data driven, offering a more objective and evidence-based alternative to traditional methods.^13^ This paradigm shift allows for the customization of treatment plans to align with each patient’s unique risk profile. Accordingly, we developed a web-based tool leveraging demographic and multiple clinical variables to ascertain the probability of PEP.

Our online prediction tool demonstrates high accuracy in both internal and external validation datasets, but its practical use in real clinical settings remains an important consideration. Preliminary data suggest that the tool offers valuable guidance for clinical decision-making. However, to confirm its effectiveness and broader applicability, these findings need to be validated through multicenter real-world testing. Moving forward, we plan to refine and enhance the tool based on clinician feedback, ensuring it is more broadly applicable and useful in clinical practice.

In summary, our study utilized six machine learning algorithms to accurately predict the risk of PEP, incorporating 25 parameters encompassing demographic and various clinical variables in patients who achieved complete clearance of CBDSs via ERCP. Through PDP, we were able to discern non-linear relationships between critical indicators such as age, the diameter of the common bile duct, stone diameter, and the associated risk of PEP. Notably, the RF algorithm demonstrated superior accuracy in predicting PEP risk, as confirmed by both internal and external validation. The development of a visual network representation of this algorithm presents a significant advancement. It would empower clinicians to more effectively assess the risk of PEP in patients with choledocholithiasis post-complete stone clearance. This tool would facilitate the formulation of proactive, individualized treatment strategies, thereby enhancing patient care and outcomes in the context of ERCP procedures.

### Limitations of the study

In the ERCP procedures assessed, specifics such as the duration of balloon dilation and the choice of stone removal instrument (basket, balloon, or both) were not comprehensively detailed, and the use of mechanical lithotripsy was not accounted for. Additionally, the expertise of the endoscopist, which is a well-recognized influencing factor in ERCP outcomes, was not systematically evaluated. These procedural factors can affect both the success of stone removal and the risk of complications such as PEP. Furthermore, retrospective studies, essential for analyzing pre-existing data, inherently face limitations, such as data quality and completeness issues, selection bias, and challenges in controlling for confounding variables. Furthermore, in the context of external validation datasets, the inclusion of a small number of cases from certain centers can affect the study’s generalizability, reduce its statistical power, and limit the representation of diverse clinical settings. To further refine and validate our findings, future studies should focus on multicenter, large-scale prospective research in the context of PEP. Such efforts will enhance the robustness and applicability of our findings, contributing to improved patient outcomes in ERCP-related procedures.

## Resource availability

### Lead contact

Further information and requests for resources should be directed to the lead contact, Cheng Wang (wangc1203@ustc.edu.cn).

### Materials availability

This study did not generate new unique reagents.

### Data and code availability

• The data cannot be made publicly accessible due to hospital regulations. Distributing these data without the necessary consent could potentially breach patient confidentiality and contravene the approval granted by the Institutional Review Board for this study. Any additional information required to reanalyze the data reported in this paper is avail-able from the lead contact on request.
• All original code has been deposited at https://github.com/jamesjin63/machine-learning-in-PEP and is publicly available as of the date of publication.
• Any additional information required to reanalyze the data reported in this paper is available from the lead contact upon request.

## Acknowledgments

This study was supported by the 10.13039/501100001809National Natural Science Foundation of China for Young Scholar (no. 82203856) and the Key Research and Development Project Foundation of Anhui Province (no. 2022e07020006).

## Author contributions

Conceptualization, design, and methodological framework, G.W., Q.S., H.Z., S.Q., and C.W.; patient inclusion, exclusion, and data acquisition, P.X., Xiangyu He, Xiangkun He, Xiaosi Hu, M.S., Q.Z., Z.F., Y.C., Y.G., Z.J., W.L., H.T., and C.Y.; secondary review of patient inclusion and data collection, Y.W., S.X., G.H., and X.Z.; interpreting the relevant imaging data, Y.W. and S.X.; statistical analysis, logical interpretation, and results presentation, G.W. and J.Z.; composing initial drafts and editing tables and figures, G.W., Q.S., and S.Q.

## Declaration of interests

The authors declare no competing interests.

## STAR★Methods

### Key resources table

**Table:** 

REAGENT or RESOURCE	SOURCE	IDENTIFIER
**Deposited data**		

Raw Electronic Medical Record data	This paper	N/A
Online web calculations page	This paper	https://jamesjin63.shinyapps.io/Shiny_PEP_USTC/

**Software and algorithms**		

RStudio	Posit team (2023)	http://www.posit.co/
Shiny (R package)	Posit team (2023)	https://shiny.posit.co/
Caret (R package)	Max Kuhn (2019)	https://topepo.github.io/caret/
ggplot2 (R package)	Hadley Wickham (2024)	https://github.com/tidyverse/ggplot2
dplyr (R package)	Hadley Wickham (2024)	https://github.com/tidyverse/dplyr

### Experimental model and study participant details

The retrospective cohort study aimed to develop and validate a pancreatitis-specific risk model for patients with choledocholithiasis following complete ERCP stone clearance. The study encompassed two pivotal phases: the development of the model internally and its subsequent external validation. Data for the initial model development and internal validation were sourced from the clinical records of 2955 patients who underwent ERCP for common bile duct stones at the First Affiliated Hospital of USTC between January 2015 and January 2023. From this cohort, cases involving concurrent pancreatic duct stones, acute cholangitis, unsuccessful stone removal, residual stones, unsuccessful procedures, or incomplete data were systematically excluded to ensure the homogeneity of the study and avoid introducing confounding variables due to these complex conditions (as depicted in Graphical abstract). None of the patients were routinely treated with rectal NSAIDs or hydration with lactated Ringer’s solution, as the focus of our study was on identifying risk factors and developing a predictive model, rather than evaluating the impact of these preventive interventions. Consequently, a total of 2247 cases constituted the internal dataset for the study. For external validation, the dataset comprised clinical data from patients meeting the aforementioned criteria across 12 centers (A-L) in China, recorded from January 2021 to January 2023. The 12 centers included 53 cases from Northern Jiangsu People’s Hospital (Center A), 53 from Hunan Provincial People’s Hospital (Center B), 58 from No. 904 Hospital of the PLA Joint Logistics Support Force (Center C), 56 from the First Affiliated Hospital of Anhui Medical University (Center D), 20 from Anhui No.2 Provincial People’s Hospital (Center E), 52 from the First Affiliated Hospital of Bengbu Medical College (Center F), 119 from the Second People’s Hospital of Hefei (Center G), 73 from the Second Affiliated Hospital of Nanjing Medical University (Center H), 50 from the Second Affiliated Hospital of Soochow University (Center I), 62 from Xuzhou Central Hospital (Center J), 49 from the First Affiliated Hospital of Wannan Medical College (Center K), and 29 from the First People’s Hospital of Hefei (Center L), culminating in a total of 674 cases from the above 12 centers. The study received approval from the hospital’s ethics committee (No. 2023-RE-207; Jan 20, 2023) and was conducted in strict adherence to the Helsinki Declaration guidelines. Given its retrospective nature, the requirement for informed consent was duly waived.

For the purposes of model development and validation, the internal dataset was stratified into a training set, constituting 80% of the cohort (*n* = 1798), and a test set, comprising the remaining 20% (*n* = 449). This division was meticulously executed through random sampling, ensuring that no statistically significant differences in clinical characteristics were present between the two groups, thereby maintaining the integrity and reliability of the model. This stratification process and the resultant homogeneity in clinical features across both datasets are detailed in Table S2.

### Method details

#### Data extraction and definitions

All ERCP procedures in this study were meticulously performed in alignment with standardized norms to minimize potential biases. Comprehensive patient data were collected, encompassing demographics (gender, age, height, weight), medical history (hypertension, diabetes mellitus, history of pancreatitis, history of hepatitis), and preoperative evaluations, including serological tests (white blood cell [WBC] count, neutrophil [N] count, hemoglobin [HB], platelet [PLT] count, total bilirubin [TB], alanine transaminase [ALT] and aspartate transaminase [AST] levels) and imaging assessments (diameter of common bile duct, diameter of stone, gallbladder status, presence of gallstones or intrahepatic bile duct stones, stone quantity). Surgical variables were also recorded, such as the anatomical relationship between the papillae and diverticula, the difficulty of cannulation, and the application of endoscopic sphincterotomy (EST) or endoscopic papillary balloon dilation (EPBD). Post-ERCP complications were meticulously documented. The primary objective of the study was to predict the risk of developing PEP. "Difficult cannulation" was specifically defined as necessitating more than five attempts, extending beyond 5 min, or inadvertent pancreatic duct cannulation on more than three occasions.^14^ The criteria for diagnosing pancreatitis conformed to established consensus guidelines.^15^ A diagnosis of PEP was made in cases where patients exhibited new or exacerbated pancreatic-type abdominal pain persisting for at least 24 h post-procedure, accompanied by a serum amylase level exceeding three times the normal upper limit. Complete stone clearance was verified through a concluding cholangiogram.

#### Model interperation

In this study, variables demonstrating statistical significance (*p* < 0.05) in univariate logistic regression analysis were selected for inclusion in the multivariate logistic regression model. The multivariate analysis aimed to identify independent risk factors for PEP, with a significance threshold set at *p* < 0.05. To ascertain the incremental influence of each variable on the model’s predictive capacity, Partial Dependence Plots (PDPs) were employed, available through the PDP package (https://cran.r-project.org/web/packages/pdp/).^16^ These plots facilitate an understanding of the relationship between clinical characteristics and the risk of PEP, considering the averaged impact of other variables in the model. The selection criteria for potential candidate variables were based on *p* less than 0.05 or 0.10 in the univariate analysis. Utilizing the PDP package, the study further explored the interactions between pairs of factors, maintaining other model variables constant. This approach allowed for a nuanced visualization of how varying one factor affects the model’s response, thereby enhancing the interpretability and applicability of the multivariate logistic regression model in assessing PEP risk.

#### Machine learning algorithm

Several machine learning models are compared to select the best classifier for PEP prediction. In addressing the binary classification challenge, a comprehensive assessment was conducted using six distinct machine learning algorithms: eXtreme Gradient Boosting (XGBoost), Support Vector Machine (SVM), Random Forest (RF), Naive Bayes (NB), Generalized Linear Models (GLM), and Decision Trees (DT). The clinical characteristics from the training set were used as the independent variables to develop ML models for predicting PEP with 5-fold cross-validation in order to avoid redundant information in train dataset. In the refinement of each machine learning algorithm (XGBoost, SVM, RF, NB, GLM, DT), an extensive hyperparameter tuning exercise was conducted to optimize predictive performance within the training dataset. This process involved the implementation of various established methods, including Grid Search, Random Search, and Bayesian Hyperparameter Optimization. Grid Search methodically traversed all possible parameter combinations, Random Search offered a probabilistic approach to parameter selection, and Bayesian Optimization utilized Bayesian principles for a more targeted search within the parameter space. Significantly, the methodologies and specific parameters utilized in this hyperparameter optimization process have been meticulously documented and are publicly accessible on https://github.com/jamesjin63/machine-learning-in-PEP. This transparency allows for replication and further exploration of our methods by the broader scientific community. The risk of PEP was predicted in the internal and external test dataset and the predictive performance was evaluated. Given the presence of class imbalance in the data, the Synthetic Minority Over-sampling Technique was employed to generate synthetic instances within the training dataset, thereby enhancing the representation of minority class features. Model performance was subsequently evaluated using the Area Under the Receiver Operating Characteristic Curve (AUC). Additionally, the sensitivity and specificity of the machine learning algorithms were rigorously quantified in both the internal and external test datasets.

#### Online web application

Subsequent to the validation phase, the model demonstrating optimal performance, incorporating numerous clinical indicators, was selected to underpin a web-based risk calculator. This interactive tool, devised for the prediction of PEP risk in patients with choledocholithiasis, was developed using the 'shiny' package in R.

### Quantification and statistical analysis

The clinical characteristics from the training set were used as the independent variables to develop ML models for predicting PEP with 5-fold cross-validation in order to avoid redundant information in train dataset. In the refinement of each machine learning algorithm (XGBoost, SVM, RF, NB, GLM, DT), an extensive hyperparameter tuning exercise was conducted to optimize predictive performance within the training dataset. This process involved the implementation of various established methods, including Grid Search, Random Search, and Bayesian Hyperparameter Optimization.

Statistical analyses in this study were conducted using R software (version 4.0.2; http://www.R-project.org) and Python (version 3.10.9: https://www.python.org/). For continuous variables adhering to a normal distribution, a two-sided paired t-test was employed to assess differences between groups. In instances where continuous variables deviated from normal distribution, the Mann-Whitney U test was utilized for comparisons. For categorical variables, Pearson’s chi-squared (χ^2^) test was applied to evaluate the differences in frequency distributions between the two groups, ensuring robust and appropriate statistical assessment of the data. Statistical significance was defined as *p* < 0.05.
