# Supplementary material for: Development and external validation of a model for post-endoscopic retrograde cholangiopancreatography pancreatitis
Source: iScience. 2025 May 2;28(6):112570. doi: 10.1016/j.isci.2025.112570 (PMC12146526; doi:10.1016/j.isci.2025.112570)
Supplement: Document S1. Tables S1 and S2 [file mmc1.pdf]

iScience, Volume *28*

Supplemental information

Development and external validation of a model for post-endoscopic retrograde cholangiopancreatography pancreatitis

Gang Wang, Qikai Sun, Hai Zhu, Song Qiao, Peng Xu, Xiangyu He, Xiangkun He, Xiaosi Hu, Mingming Song, Qiuyan Zhang, Zhenyu Feng, Yue Chen, Yue Gao, Zhiyuan Jin, Wen Li, Haizheng Tang, Chaoqun Yan, Yajun Wei, Shibo Xu, Gang Hu, Xiuhua Zhang, Jinxin Zheng, and Cheng Wang

Models	XGBOOST	SVM	RF	NB	GLM	DT
A	0.813 (0.531-1.000)	0.673 (0.460-0.885)	0.740 (0.240-1.000)	0.393 (0.020-0.979)	0.713 (0.288-0.980)	0.743 (0.402-0.951)
B	0.804 (0.527-0.983)	0.575 (0.333-0.853)	0.848 (0.637-0.985)	0.825 (0.475-1.000)	0.804 (0.631-0.929)	0.685 (0.400-0.910)
C	0.636 (0.322-0.921)	0.479 (0.242-0.728)	0.759 (0.500-0.947)	0.782 (0.542-1.000)	0.812 (0.537-1.000)	0.555 (0.392-0.761)
D	0.556 (0.241-0.870)	0.454 (0-0.927)	0.653 (0.273-0.981)	0.630 (0.200-1.000)	0.694 (0.296-1.000)	0.343 (0.274-0.404)
E	1.000 (1.000-1.000)	0	0.895 (0.737-1.000)	0.053 (0-0.167)	0.947 (0.833-1.000)	0.395 (0.306-0.474)
F	0.890 (0.786-0.979)	0.790 (0.627-0.922)	0.810 (0.520-1.000)	1.000 (1.000-1.000)	1.000 (1.000-1.000)	0.950 (0.908-0.990)
G	0.569 (0.403-0.729)	0.452 (0.292-0.621)	0.576 (0.417-0.732)	0.441 (0.264-0.627)	0.646 (0.464-0.813)	0.522 (0.395-0.666)
H	0.635 (0.437-0.840)	0.686 (0.526-0.827)	0.768 (0.613-0.909)	0.764 (0.550-0.931)	0.783 (0.669-0.898)	0.610 (0.458-0.774)
I	0.630 (0.220-1.000)	0.755 (0.184-1.000)	0.598 (0.214-0.959)	0.826 (0.612-1.000)	0.777 (0.583-0.984)	0.538 (0.370-0.844)
J	0.779 (0.623-0.917)	0.439 (0.108-0.810)	0.882 (0.733-0.989)	0.821 (0.561-1.000)	0.860 (0.672-0.994)	0.739 (0.448-0.958)
K	0.855 (0.638-1.000)	0.558 (0.426-0.691)	0.913 (0.750-1.000)	0.920 (0.833-0.986)	0.920 (0.787-1.000)	0.359 (0.287-0.417)
L	0.650 (0.330-0.974)	0.530 (0.111-1.000)	0.750 (0.462-1.000)	0.770 (0.214-1.000)	0.780 (0.286-1.000)	0.590 (0.304-0.875)

Table S1. Prediction performance of six machine learning algorithms in 12 external central datasets.

Variables		All patients (2247)	Training set (1798)	Validation set (449)	*P*
**Gender**	Male	1054 (46.91)	849 (47.22%)	205 (45.66%)	.589
	Female	1193 (53.09)	949 (52.78%)	244 (54.34%)	
**Age (year)**		59.88 (15.96)	60.16 (15.93)	58.77 (16.01)	.099
**Height (cm)**		163.04 (8.46)	162.95 (8.51)	163.38 (8.24)	.336
**Weight (kg)**		62.02 (11.19)	61.96 (11.14)	62.24 (11.40)	.640
**HBP**	NO	1560 (69.43)	1240 (68.97%)	320 (71.27%)	.373
	YES	687 (30.57)	558 (31.03%)	129 (28.73%)	
**DM**	NO	1961 (87.27)	1566 (87.10%)	395 (88.97%)	.675
	YES	286 (12.73)	232 (12.90%)	54 (12.03%)	
**History of pancreatitis**	NO	2125 (94.57)	1702 (94.66%)	423 (94.21%)	.794
	YES	122 (5.43)	96 (5.34%)	26 (5.79%)	
**History of hepatitis**	NO	2198 (97.82)	1759 (97.83%)	439 (97.77%)	1.000
	YES	49 (2.18)	39 (2.17%)	10 (2.23%)	
**WBC (10^9 / L)**		6.04 (2.72)	6.04 (2.73)	6.04 (2.70)	.983
**N (10^9 / L)**		3.87 (2.65)	3.87 (2.66)	3.89 (2.63)	.839
**HB (g / L)**		127.98 (16.62)	127.77 (16.79)	128.81 (15.94)	.237
**PLT (10^9 / L)**		203.92 (75.56)	203.57 (76.14)	205.32 (73.27)	.662
**TB (umol / L)**		35.33 (49.65)	36.03 (49.88)	32.51 (48.69)	.178
**ALT (U / L)**		121.31 (169.47)	121.90 (171.90)	118.96 (159.51)	.742
**AST (U / L)**		85.00 (129.48)	85.00 (129.66)	84.98 (128.89)	.996
**Diameter of common bile duct (mm)**		10.86 (4.40)	10.88 (4.42)	10.78 (4.34)	.667
**Diameter of the largest stone (mm)**		7.18 (3.59)	7.19 (3.57)	7.16 (3.65)	.860
**Presence of the gallbladder**	NO	1369 (60.93)	1088 (60.51%)	281 (62.58%)	.453
	YES	878 (39.07)	710 (39.49%)	168 (37.42%)	
**With gallstone**	NO	1630 (72.54)	1301 (72.36%)	329 (73.27%)	.742
	YES	617 (27.46)	497 (27.64%)	120 (26.73%)	
**With intrahepatic bile duct stones**	NO	2061 (91.72)	1646 (91.55%)	415 (92.43%)	.610
	YES	186 (8.28)	152 (8.45%)	34 (7.57%)	
**Number of Stones**	1	956 (42.55)	759 (42.21%)	197 (43.88%)	.816
	2	148 (6.59)	119 (6.62%)	29 (6.46%)	
	multiple	1143 (50.87)	920 (51.17%)	223 (49.67%)	
**Position relationship between papilla and diverticula**	In	103 (4.58)	90 (5.01%)	13 (2.90%)	.149
	Next	675 (30.04)	541 (30.09%)	134 (29.84%)	
	Non-diverticula	1469 (65.38)	1167 (64.91%)	302 (67.26%)	
**Difficult cannulation**	NO	2079 (92.52)	1667 (92.71%)	412 (91.76%)	.557
	YES	168 (7.48)	131 (7.29%)	37 (8.24%)	
**EST**	NO	1273 (56.65)	1029 (57.23%)	244 (54.34%)	.293
	YES	974 (43.35)	769 (42.77%)	205 (45.66%)	
**EPBD**	NO	555 (24.7)	432 (24.03%)	123 (27.39%)	.156
	YES	1692 (75.30)	1366 (75.97%)	326 (72.61%)	
**PEP**	NO	2171 (96.62)	1737 (96.61%)	434 (96.66%)	1.000
	YES	76 (3.38)	61 (3.39%)	15 (3.34%)	

Table S2. Clinical data of training and validation data set in patients with CBDs.
